# A polymorphism in the CYP17 gene is associated with male breast cancer

**DOI:** 10.1038/sj.bjc.6690663

**Published:** 1999-09

**Authors:** I E Young, K M Kurian, C Annink, I H Kunkler, V A Anderson, B B Cohen, M L Hooper, A H Wyllie, C M Steel

**Affiliations:** Sir Alastair Currie CRC Laboratories, University of Edinburgh Department of Pathology, , Molecular Medicine Centre and; Department of Clinical Oncology, Western General Hospital, Crewe Road, Edinburgh, EH4 2XU, UK; School of Biomedical Sciences, Bute Medical Building St Andrews, Fife, KY16 9TS, UK; Department of Biology & Medical Laboratory Education, University of Professional Education Enschede, Postbus 70000, Enschede, 7500 KB, The Netherlands; Department of Pathology, University of Cambridge, Tennis Court Road, Cambridge, CB2 1QP, UK

**Keywords:** CYP17 polymorphism, male breast cancer

## Abstract

The CYP17 gene codes for the cytochrome P450c17α enzyme that is involved in the synthesis of oestrogens. This case-control study from the South East of Scotland shows that a polymorphism of the CYP17 gene is associated with an increased risk of male breast cancer. © 1999 Cancer Research Campaign


*British Journal of Cancer* (1999) **81**(1), 141–143
© 1999 Cancer Research Campaign
Article no. bjoc.1999.0663

Short Communication

### A polymorphism in the CYP17 gene is associated with male breast cancer

**IE Young[1], KM Kurian[1], C Annink[1,4], IH Kunkler[2], VA Anderson[3], BB Cohen[3], ML Hooper[1], AH Wyllie[1,5] and CM Steel[3]**

[1]Sir Alastair Currie CRC Laboratories, University of Edinburgh Department of Pathology, Molecular Medicine Centre and [2]Department of Clinical Oncology, Western General Hospital, Crewe Road, Edinburgh EH4 2XU, UK; [3]School of Biomedical Sciences, Bute Medical Building, University of St Andrews, St Andrews, Fife KY16 9TS, UK; [4]Department of Biology & Medical Laboratory Education, University of Professional Education Enschede, Postbus 70000, 7500 KB Enschede, The Netherlands; [5]Department of Pathology, University of Cambridge, Tennis Court Road, Cambridge CB2 1QP, UK

**Summary** The CYP17 gene codes for the cytochrome P450c17α enzyme that is involved in the synthesis of oestrogens. This case-control study from the South East of Scotland shows that a polymorphism of the CYP17 gene is associated with an increased risk of male breast cancer.

**Keywords**: CYP17 polymorphism; male breast cancer

Mutations of two tumour suppressor genes, BRCA1 and BRCA2, have been identified in some cases of familial and early onset breast cancer (Miki et al, 1994; Wooster et al, 1995). These mutations, however, account for no more than about 5% of total cases of female breast cancer. Male breast cancer is rare (approximately 1% of all cases of breast cancer) and therefore less is known about the genetic influences in its development. Male breast cancer has been linked to mutations of the BRCA2 gene in some cases. The frequency of mutations in the series studied varies widely, from 4% to 40% (Thorlacius et al, 1996; Friedman et al, 1997).

It has been suggested there may be other genetic factors that confer a relatively low absolute risk to the individual, but potentially could result in a substantial number of cases in the whole population (Feigelson et al, 1996). The CYP17 gene on chromosome 10 codes for the cytochrome P450c17α enzyme that catalyses steroid 17α-hydroxylase and 17,20-lyase activities (Picado-Leonard, 1987). It is therefore a key regulator in the synthesis of androgens and oestrogens from their steroid precursors (Brentano et al, 1990). A polymorphic T to C substitution has been described that creates an additional CCACC type promoter site 34 bp upstream from the site of initiation of translation (Carey et al, 1994). It is thought that the additional promoter site may increase the rate of transcription of the gene and thereby increase enzyme activity. Serum oestradiol levels are higher in women hetero- and homozygous for the C allele of the CYP17 gene (Feigelson et al, 1998). One study found that the C allele was associated with an increased risk of advanced breast cancer (Feigelson et al, 1997). A larger and more recent study, however, found no such association (Dunning et al, 1998). Male breast cancer is known to be associated with conditions, for example Klinefelter's syndrome, that result in increased levels of serum oestrogens (Jackson et al, 1965). Mutation of the androgen receptor gene has also been reported in a few cases of male breast cancer (Wooster et al, 1992; Lobaccaro et al, 1993). This mutation results in a change in the androgen–oestrogen balance (low levels of androgens, increased levels of oestrogens). Given that the C allele of the CYP17 gene is associated with increased serum oestradiol levels, then it could also be implicated in the development of male breast cancer. The aim of this study was to test this hypothesis.

*Received 12 January 1999*
*Revised 10 February 1999*
*Accepted 12 February 1999*

*Correspondence to:* IE Young

### METHODS

### Case and control population selection

Male cases were taken from a consecutive series of 76 male breast cancer patients treated in the South East of Scotland between 1974 and 1998. These patients ranged in age from 26 to 91 years at the time of diagnosis. Living patients were contacted through their General Practitioners. Initial contact was by telephone where possible. Peripheral blood samples (10 ml) were obtained from 24 living male breast cancer patients. Written informed consent was obtained in all cases. Archival wax-embedded tissue sections were obtained for 39 deceased patients. DNA previously extracted from a blood sample and stored was available for one of the deceased patients. Two patients declined to take part in the study. Archival specimens were not available in ten cases. Female cases were a consecutive group of 39 patients with early onset (age < 50 years) breast cancer referred to the Edinburgh Breast Unit, Western General Hospital, Edinburgh between 1987 and 1990.

Male and female control DNA samples were extracted from blood donations to the Edinburgh and South East Scotland Blood Transfusion service, from placental tissue and blood donations to the School of Biological and Medical Sciences, University of St Andrews, and from a small number of tonsillectomy specimens provided by the Department of ENT Surgery, City Hospital, Edinburgh. We believe that these samples are representative of the population within the South East Scotland region.

### Laboratory methods

DNA extraction was from whole blood by standard phenol–chloroform extraction. DNA extraction from wax-embedded tissue was from 10-μm sections incubated at 55°C with a lysis buffer and proteinase K.

The following primers were designed for polymerase chain reaction (PCR): CYP17S-F, 5′-CAAAAGTCAAGGTGAA-GATCAG-3′; CYP17S-R, 5′-TAGGGTAAGCAGCAAGAGAG-3′. These generate PCR fragments 150 base pairs in length, including the polymorphic site. PCR reactions were performed in 50-μl aliquots, each containing 1 × buffer, 2 mM magnesium chloride, 100 μM deoxynucleoside triphosphates, 100 pmol of each primer, 1 unit of *Taq* polymerase (Life Technologies™) and approximately 100 ng DNA. The amplification was performed using an OmniGene thermal cycler (Hybaid, UK) under the following conditions: initial denaturation at 94°C for 3 min; amplification for 38 cycles, with denaturation at 94°C for 45 s, annealing at 56°C for 45 s and extension at 72°C for 45 s; final extension at 72°C for 10 min. The PCR products were digested with MspA1 (Promega, Madison, WI, USA) for 90 min at 37°C. The products were separated by electrophoresis on a 4% agarose gel, staining with ethidium bromide and identified from photographs of the gel under ultraviolet illumination.

### Data analysis

The association between the C allele of the CYP17 gene and breast cancer in the male and female subjects and controls was analysed using $\chi^2$ tests.

### RESULTS

The results showing the genotyping of the cases and controls are shown in Table 1. The results show that a C allele is found significantly more frequently in male breast cancer patients than in male controls ($\chi^2 = 4.308$, 1 d.f., $P = 0.038$). The odds ratio (OR) for the risk of a male with a C allele developing breast cancer is calculated as 2.10 (95% confidence interval (CI) 1.04–4.27). There were no significant differences between female breast cancer patients and female controls ($\chi^2 = 1.895$, 1 d.f., $P = 0.169$; OR = 0.56, 95% CI 0.25–1.28). The distribution of alleles in both control groups is in Hardy–Weinberg equilibrium (Table 2).

### DISCUSSION

To our knowledge, this is the first study that attempts to evaluate the possible role of a polymorphism of the CYP17 gene in the development of male breast cancer. We have found that the polymorphic T to C substitution within the CYP17 gene occurs significantly more frequently in male breast cancer patients than in male controls. These results are consistent with other reports that there may be a hormonal contribution to the development of male breast cancer (Jackson et al, 1965; Lobaccaro et al, 1993; Wooster et al, 1992). We have also studied a small group of female breast cancer patients from the same geographical region. We found no association between the presence of a C allele of the CYP17 gene and female breast cancer. The first study that looked at this polymorphism in female breast cancer patients (Feigelson et al, 1997), found a significant association only after sub-selecting those patients that presented with advanced disease. The second, and much larger, study (Dunning et al, 1998) did find a small increase in the risk of breast cancer associated with the C allele, but this increase was not statistically significant. It is possible that the T to C polymorphism in the CYP17 gene is associated with higher levels of serum oestrogens, but that because the endogenous oestrogen level is higher in females than in males, further increase does not have a significant effect. The association between an increase in the levels of serum oestrogens and the development of breast cancer may therefore be detectable only in males, or perhaps as previously suggested (Dunning et al, 1998), among post-menopausal females.

Further studies of this type are required, specifically to test for any association between the polymorphism of the CYP17 gene and levels of serum oestrogens in males.

**Table 1** CYP17 genotype frequencies among breast cancer patients and controls

| | Males | | Females | |
|---|---|---|---|---|
| **Genotype** | **Cases (*n* = 64)** | **Controls (*n* = 81)** | **Cases (*n* = 39)** | **Controls (*n* = 58)** |
| TT | 17 (26.6%) | 35 (43.2%) | 21 (53.8%) | 23 (39.7%) |
| TC | 42 (65.6%) | 39 (48.1%) | 13 (33.3%) | 28 (48.3%) |
| CC | 5 (7.8%) | 7 (8.6%) | 5 (12.8%) | 7 (12.1%) |
| TC/CC | 47 (73.4%) | 46 (56.8%) | 18 (46.2%) | 35 (60.3%) |

**Table 2** CYP17 genotype frequencies in controls and expected frequencies under Hardy–Weinberg equilibrium

| | Male controls | | Female controls | |
|---|---|---|---|---|
| | **Observed** | **Expected** | **Observed** | **Expected** |
| TT | 35 | 36.7 | 23 | 23.6 |
| TC | 39 | 35.7 | 28 | 26.8 |
| CC | 7 | 8.67 | 7 | 7.60 |
| | ($\chi^2 = 0.705$, 2 d.f., $P = 0.703$) | | ($\chi^2 = 0.116$, 2 d.f., $P = 0.943$) | |

© Cancer Research Campaign 1999

### ACKNOWLEDGEMENTS

We thank Mr R Morris and Dr S Bader for technical advice; Dr T Anderson, Dr A McGregor, Dr I Nawroz, Dr K Ramesar and Dr AM Lutfy for making available the archival wax-embedded tissue sections; and Miss G Kerr and the medical records staff in the Department of Clinical Oncology, Western General Hospital, Edinburgh. We would like to thank Staff in the Department of Blood Transfusion Medicine, Royal Infirmary of Edinburgh and in the Department of ENT Surgery, City Hospital, Edinburgh for providing some of the control samples. This work has been funded by grants from the Royal College of Surgeons of Edinburgh, the Sarah Percy Fund, the Melville Trust for the Care and Cure of Cancer and the Robertson Trust.

### REFERENCES

Brentano ST, Picado-Leonard J, Mellon SH, Moore CCD and Miller WL (1990) Tissue-specific, cyclic adenosine 3′,5′ -monophosphate-induced, and phorbol ester-repressed transcription from the human P450c17 promoter in mouse cells. *Mol Endocrinol* **4**: 1972–1979

Carey AH, Waterworth D, Patel K, White D, Little J, Novelli P, Franks S and Williamson R (1994) Polycystic ovaries and premature male pattern baldness are associated with one allele of the steroid metabolism gene CYP17. *Hum Mol Genet* **3**: 1873–1876

Dunning AM, Healey CS, Pharoah PDP, Foster NA, Lipscombe JM, Redman KL, Easton DF, Day NE and Ponder BAJP (1998) No association between a polymorphism in the steroid metabolism gene Cyp17 and risk of breast cancer. *Br J Cancer* **77**: 2045–2047

Feigelson HS, Ross RK, Yu MC, Coetzee GA, Reichardt JKV and Henderson BE (1996) Genetic susceptibility to cancer from exogenous and endogenous exposures. *J Cell Biochem* Suppl **25**: 15–22

Feigelson HS, Coetzee GA, Kolonel LN, Ross RK and Henderson BE (1997) A polymorphism in the CYP17 gene increases the risk of breast cancer. *Cancer Res* **57**: 1063–1065

Feigelson HS, Shames LS, Pike MC, Coetzee GA, Stanczyk FZ and Henderson BE (1998) Cytochrome P450c17α gene (CYP17) polymorphism is associated with serum estrogen and progesterone concentrations. *Cancer Res* **58**: 585–587

Friedman LS, Gayther SA, Kurosaki T, Gordon D, Noble B, Casey G, Ponder BAJP and Anton-Culver H (1997) Mutation analysis of BRCA1 and BRCA2 in a male breast cancer population. *Am J Hum Genet* **60**: 313–319

Jackson AW, Muldal S, Ockey CH and O'Connor PJ (1965) Carcinoma of the male breast in association with the Klinefelter syndrome. *Br Med J* **1**: 223–225

Lobaccaro JM, Lumbroso S, Belon C, Galtier-Dereure F, Bringer J, Lesimple T, Heron JF, Pujol H and Sultan C (1993) Male breast cancer and the androgen receptor gene (Letter). *Nat Genet* **5**(2): 109–110

Miki Y, Swensen J, Shattuck-Eidens D, Futreal PA, Harshman K, Tavtigian S, Liu Q, Cochran C, Bennett LM, Ding W, Bell R, Rosental J, Hussey C, Tran T, McClure M, Frye C, Hattier T, Phelps R, Haugen-Strano R, Katcher H, Gholami Z, Shaffer D, Stone S, Bayer S, Wray C, Bogden R, Dayananth P, Ward J, Tonin P, Narod S, Bristow PK, Norris FH, Helvering L, Morrison P, Rosteck P, Lai M, Barrett JC, Lewis C, Neuhausen S, Cannon-Albright L, Goldgar D, Wiseman R, Kamb A and Skolnick MHA (1994) A strong candidate for the breast and ovarian cancer susceptibility gene BRCA1. *Science* **266**: 66–71

Picado-Leonard J and Miller WL (1987) Cloning and sequence of the human gene for P450c17 (steroid 17α-hydroxylase/17,20-lyase): similarity with the gene for P450c21. *DNA* **6**(5): 439–448

Thorlacius S, Olafsdottir G, Tryggvadottir L, Neuhausen S, Jonasson JG, Tavtigian S, Tulinius H, Ögmundsdottir HM and Eyfjörd JE (1996) A single BRCA2 mutation in male and female breast cancer families from Iceland with varied cancer phenotypes. *Nat Genet* **13**: 117–119

Wooster R, Mangion J, Eeles R, Smith S, Mitchell D, Averill D, Barrett-Lee P, Easton DF, Ponder BAJP and Stratton MR (1992) A germline mutation in the androgen receptor gene in two brothers with breast cancer and Reifenstein syndrome. *Nat Genet* **2**(2): 132–134

Wooster R, Bignell G, Lancaster J, Swift S, Seal S, Mangion J, Collins N, Gregory S, Gumbs C, Micklem G, Barfoot R, Hamoudi R, Patel S, Rice C, Biggs P, Hashim Y, Smith A, Connor F, Arason A, Gudmundsson J, Ficenec D, Kelsell D, Ford D, Tonin P, Bishop DT, Spurr NK, Ponder BAJP, Eeles R, Peto J, Devilee P, Cornelisse C, Lynch H, Narod S, Lenoir G, Egilsson V, Barkadottir RB, Easton DF, Bentley DR, Futreal PA, Ashworth A and Stratton MR (1995). Identification of the breast cancer susceptibility gene BRCA2. *Nature* **378**: 789–792

*© 1999 Cancer Research Campaign*

